# The Control of the Feeble-Minded

**Published:** 1912-07-20

**Authors:** 


### The Control of the Feeble-Minded.

Sir D. Brynmor Jones presided over the Grand Committee of the House of Commons which has entered upon the consideration of the Feeble-Minded Persons (Control) Bill. The measure, which seeks to provide for the better care and protection of feeble-minded persons, it is interesting to note, was attended at its first meeting by no Minister or representative of the Government, and a protest against the want of consideration shown by the Government led the Committee to adjourn.

Mr. Worthington Evans said there were in this country five voluntary institutions accommodating more than 2,000 persons, including some feeble-minded inmates. If a new authority were set up to deal with the feeble-minded, but not with imbeciles, there was grave danger that the working of those institutions, which were already registered and controlled by the Lunacy Commissioners, would be interfered with.

